# Social and Cognitive Impressions of Adults Who Do and Do Not Stutter Based on Listeners' Perceptions of Read-Speech Samples

**DOI:** 10.3389/fpsyg.2017.01148

**Published:** 2017-07-11

**Authors:** Lauren J. Amick, Soo-Eun Chang, Juli Wade, J. Devin McAuley

**Affiliations:** ^1^Department of Psychology, Michigan State University East Lansing, MI, United States; ^2^Department of Psychiatry, University of Michigan Ann Arbor, MI, United States

**Keywords:** social impression formation, stuttering, speech perception, cognitive ability, perceived likeability, perceived anxiety

## Abstract

Stuttering is a neurodevelopmental disorder characterized by frequent and involuntary disruptions during speech production. Adults who stutter are often subject to negative perceptions. The present study examined whether negative social and cognitive impressions are formed when listening to speech, even without any knowledge about the speaker. Two experiments were conducted in which naïve participants were asked to listen to and provide ratings on samples of read speech produced by adults who stutter and typically-speaking adults without knowledge about the individuals who produced the speech. In both experiments, listeners rated speaker cognitive ability, likeability, anxiety, as well as a number of speech characteristics that included fluency, naturalness, intelligibility, the likelihood the speaker had a speech-and-language disorder (Experiment 1 only), rate and volume (both Experiments 1 and 2). The speech of adults who stutter was perceived to be less fluent, natural, intelligible, and to be slower and louder than the speech of typical adults. Adults who stutter were also perceived to have lower cognitive ability, to be less likeable and to be more anxious than the typical adult speakers. Relations between speech characteristics and social and cognitive impressions were found, independent of whether or not the speaker stuttered (i.e., they were found for both adults who stutter and typically-speaking adults) and did not depend on being cued that some of the speakers may have had a speech-language impairment.

## Introduction

Stuttering is a speech disorder characterized by disfluent speech patterns, including irregular timing of phonemes (Di Simoni, 1974), repetitions of syllables or words, as well as frequent hesitations or pauses (Bloodstein, 1960). The life-span incidence of stuttering is estimated at 8% (Yairi and Ambrose, 2013), with ~1% prevalence among adults (Stuttering Foundation of America, 2017). Stuttering is a neurodevelopmental disorder attributed to subtle differences in brain function and anatomy affecting speech motor control (Neef et al., 2015). Cognitive and linguistic deficits may co-exist in some people who stutter, but are not considered to be causal factors in stuttering (Nippold, 2012). Likewise, significant social anxiety is present in 50–60% of adults who stutter (Blumgart et al., 2010; Craig and Tran, 2014; Iverach and Rapee, 2014), but this is understood to be the consequence, rather than the cause of stuttering, as children close to stuttering onset are not shown to differ from their fluent peers in level of anxiety (Davis et al., 2007; Alm, 2014).

It has been well documented that interactions with individuals who stutter yield negative perceptions and biases (e.g., Woods and Williams, 1976; Doody et al., 1993; Flynn and St. Louis, 2011). People who stutter are judged to be more submissive, tense, and insecure than their fluent speaking peers (Woods and Williams, 1976; Woods, 1978), as well as more guarded and nervous (Doody et al., 1993). These negative social impressions are widespread, occurring across cultures (Bebout and Arthur, 1992; Al-Khaledi et al., 2009; Abdalla and St. Louis, 2012; Przepiorka et al., 2013), and affect children (Betz et al., 2008; Langevin et al., 2009; Pachigar et al., 2011), adolescents (Blood and Blood, 2004), and adults (Hurst and Cooper, 1983). Negative social impressions of individuals who stutter are present among a diverse range of listeners, including classroom teachers, college students (Woods and Williams, 1976), and high school students (Flynn and St. Louis, 2011). While there is some evidence that previous experience with individuals who stutter reduces negative social impressions (Betz et al., 2008), others have demonstrated that biases even persist among people who are knowledgeable about stuttering, such as students in a speech and language pathology program (St. Louis and Lass, 1981), and those who have exposure or previous experience with individuals who stutter (Allard and Williams, 2008; Abdalla and St. Louis, 2012).

Stuttering and the associated negative biases have pervasive effects on the lives of individuals who stutter. Children who stutter are more vulnerable to bullying (Blood and Blood, 2004), and some teachers believe that stuttering limits students' future job prospects (Abdalla and St. Louis, 2012). Likewise, some employers believe that stuttering decreases employability (Hurst and Cooper, 1983). University students judge individuals who stutter to have lower occupational competence (Silverman and Paynter, 1990). Interestingly, many studies have shown that children and adolescents who stutter do not exhibit lower self-esteem than their fluent-speaking peers (Yovetich et al., 2000), although early work indicates some individual and situational differences in the attitudes of individuals who stutter toward their own stuttering (Johnson, 1934). Additionally, young children who stutter have more negative attitudes toward speech and communication than their peers (De Nil and Brutten, 1991).

Many researchers have provided evidence for potential sources of negative social impressions of people who stutter, and examined factors that affect these negative perceptions (Gabel, 2006; Boyle et al., 2009). Undergraduate students who express negative perceptions of people who stutter, in part attribute their attitudes to the amount of effort required to communicate with an individual who stutters (Hughes et al., 2010). Among university students, biases toward individuals who stutter can be attenuated when the listener believes that the speaker is involved in therapy, or when stuttering severity is reported to be mild (Gabel, 2006). Additionally, individuals who hold more accurate beliefs about the cause of stuttering are more likely to exhibit accommodating and helping behaviors toward individuals who stutter (Boyle et al., 2009).

What remains largely unknown is specifically how the speech characteristics of individuals who stutter affect social and cognitive impressions of the individual. In many of the previous studies that have examined social and cognitive impressions of individuals who stutter, negative impressions of individuals who stutter were generated when people were told to hypothetically *imagine* a person who stutters (Woods and Williams, 1976; Woods, 1978; St. Louis and Lass, 1981; Doody et al., 1993; Gabel, 2006). Others (e.g., Healey et al., 2007; Flynn and St. Louis, 2011) demonstrated that listeners express similar negative perceptions of individuals who stutter when they are presented with video or audio recordings of an individual speaking. However, even in these cases, listeners are informed that the speaker is an individual who stutters. Only one study that we are aware of specifically examined social impressions of individuals with speech-language impairments based on speech alone. Allard and Williams (2008) presented listeners only with audio recorded samples of speech and no information about the speaker, providing evidence that listeners judge individuals with communication disorders like stuttering to have significantly lower self-esteem and social adjustment than typical speakers. However, even in this experiment, the speaker was not a person who stutters, but rather a male actor delivering different versions of the same script to model what they judged to be the characteristics of different speech-language disorders and thus it is not known how well the actor's speech productions actually matched the speech characteristics of a person who stutters. The question addressed by the present study was whether people form negative social and/or cognitive impressions about individuals who stutter based on listening to their speech only without any additional cues or knowledge about the individuals producing the speech.

To address this question, two experiments were conducted in which naive listeners heard samples of naturally-read speech generated by adults who stutter and a control group; for each sample, listeners made a series of perceptual judgments about the speech that included social and cognitive impressions about the speaker. All of the read-speech samples were randomized and it was not known to listeners that the read speech-samples were generated by two speaker groups: adults who stutter and typically-speaking adult controls. These naturally-read speech samples were generated as part of a separate study (not reported here) that examined the effect of metronome pacing of speech on reducing disfluencies. For the present study, only the *naturally-read* speech samples without metronome pacing were used. It is important to note that the percentage of stuttering like disfluencies for *read speech* in adults who stutter is typically low and so it would not be obvious to listeners that the samples consisted of a group of speakers who stuttered and a group of speakers who did not; for the present study, the percentage of speech disfluencies for the read-speech samples from the adults who stuttered was only 2.4%, with the usual cut off to be categorized as a stuttering person is 3% stuttering-like disfluencies per 100 syllables. Thus, in the present study, the listeners who made judgments about the read-speech samples were both not provided any information about the individuals producing the samples and difference between the two groups of speakers were quite subtle. If negative social and cognitive impressions are formed based on speech cues only, then adults who stutter were predicted to be perceived to have lower cognitive ability, be less likeable, and be more anxious than typically-speaking adult controls. Moreover, for both the adults who stutter and typically-speaking adult controls, we were interested in more generally investigating the relation between perceptions of speech characteristics (fluency, naturalness, intelligibility, speech rate, and loudness) and social and cognitive impressions formed about the speakers.

## Experiment 1

### Methods

#### Participants and design

Ten undergraduate students (3 males, 7 females) participated in the experiment in return for course credit. All participants were native English speakers who were at least 18 years of age. All participants provided written informed consent prior to participating in the experiment, in accordance with the approved procedures of the Institutional Review Board of Michigan State University. The experiment implemented a single-factor (speaker type: adults who stutter vs. typically-speaking adults) within-subjects design. Participants (i.e., listeners) made judgments about thirty-two samples of naturally-read speech; half of the samples were produced by adults who stutter and half were produced by typically-speaking adults in identical conditions.

#### Stimuli

Stimuli consisted of 32 voice recordings of naturally-read speech produced by eight adults who stutter (7 males, 1 female) and eight typically-speaking adult controls (7 males, 1 female). Speakers contributing naturally-read speech samples were part of a separate study and gave consent for their speech samples to be used for research. The speakers ranged in age between 19 and 52 years (*M* = 25.1, *SD* = 9.7). There were two recordings per speaker and recordings were made using an Olympus DS-30 voice recorder. To be classified as stuttering, the speaker had to exhibit greater than 3% stuttering like disfluencies (SLD) per 100 syllables and score at least “very mild” according to the total score on the Stuttering Severity Instrument (SSI-4) (Riley and Bakker, 2009). Stuttering severity for speakers in the stuttering group ranged from mild to severe (SSI composite score range 19–37). The intra-class correlation (ICC) coefficient for two independent judges' assessments of %SLD was high (Cronbach's alpha = 0.84). The read-speech samples were readings of one of eight passages (153–169 syllables each) selected from a fourth through sixth grade reading curriculum; see Appendix. Read passages were the same for the two groups of speakers. Reading passages were balanced across speakers, such that each speaker read each passage only once and each passage was read the same number of times across speakers. For the stimulus recordings, speakers read presented passages at their usual comfortable speaking rate. The durations of the read-speech samples ranged from 31 to 139 s (*M* = 44, *SD* = 20).

#### Procedure

Participants listened to the read-speech samples over headphones and then for each sample made a series of judgments about the speech and the speaker characteristics. For each speech sample, participants rated five characteristics of the speech (fluency, naturalness, intelligibility, speech rate, and volume) and four attributes of the speaker (cognitive ability, likeability, anxiety level and likelihood of speech-language impairment); see Table 1 for the ordered list of questions and scale anchors. The same nine questions were presented to all participants after each speech sample, with all ratings ranging from one to five. Participants were not provided any additional cues, including information about the individuals who produced the speech (i.e., they were blind to who the speakers were).

**Table 1:** Ordered scale questions and scale anchors.

**Measure**	**Question**	**1**	**5**
Fluency	How fluent was the speech? Not very fluent indicates that the speech had frequent disruptions. Extremely fluent indicates that the speech was very smooth with no disruptions.	Not very fluent	Very fluent
Naturalness	How natural sounding was the speech?	Not very natural at all	Very natural
Intelligibility	How easy to understand were the words in the previous speech sample? Not very intelligible indicates that the speech was very difficult to understand. Very intelligible indicates that the speech was very easy to understand.	Not very intelligible	Very intelligible
Cognitive Ability	How would you rate the cognitive ability (intelligence) of the speaker?	Low ability	High ability
Speech-Language Impairment	How likely is the speaker to have a speech-language impairment.	Not very likely	Very likely
Likeability	How likeable did you find the speaker?	Not very likeable	Very likeable
Anxiety	How anxious sounding was the speaker?	Not anxious at all	Very anxious
Speech Rate	How slow or fast was the speech in the previous sample?	Very slow	Very fast
Volume	How loud or quiet was the speech in the previous sample?	Very quiet	Very loud

#### Apparatus

All 32 voice recordings were presented to each participant in a randomized order on a Dell PC computer using E-Prime v2.0 Professional (Psychology Software Tools, Inc.). Speech recordings were presented through Sennheiser HD 280 Pro headphones. Participants made responses to scale items using the keyboard to indicate their response on a 1–5 Likert-type scale that was presented on the computer screen.

#### Data analysis

The reliability of ratings across listeners for each of the rating measures were assessed through intra-class correlation coefficient (ICC). The ICC values ranged from α = 0.76 to α = 0.94. For the analysis, participant ratings for the two read-samples generated by each speaker were averaged. Separate within-subjects ANOVAs were then conducted for each dependent measure, comparing adults who stutter to typically-speaking adults.

### Results and discussion

**Figure 1:**
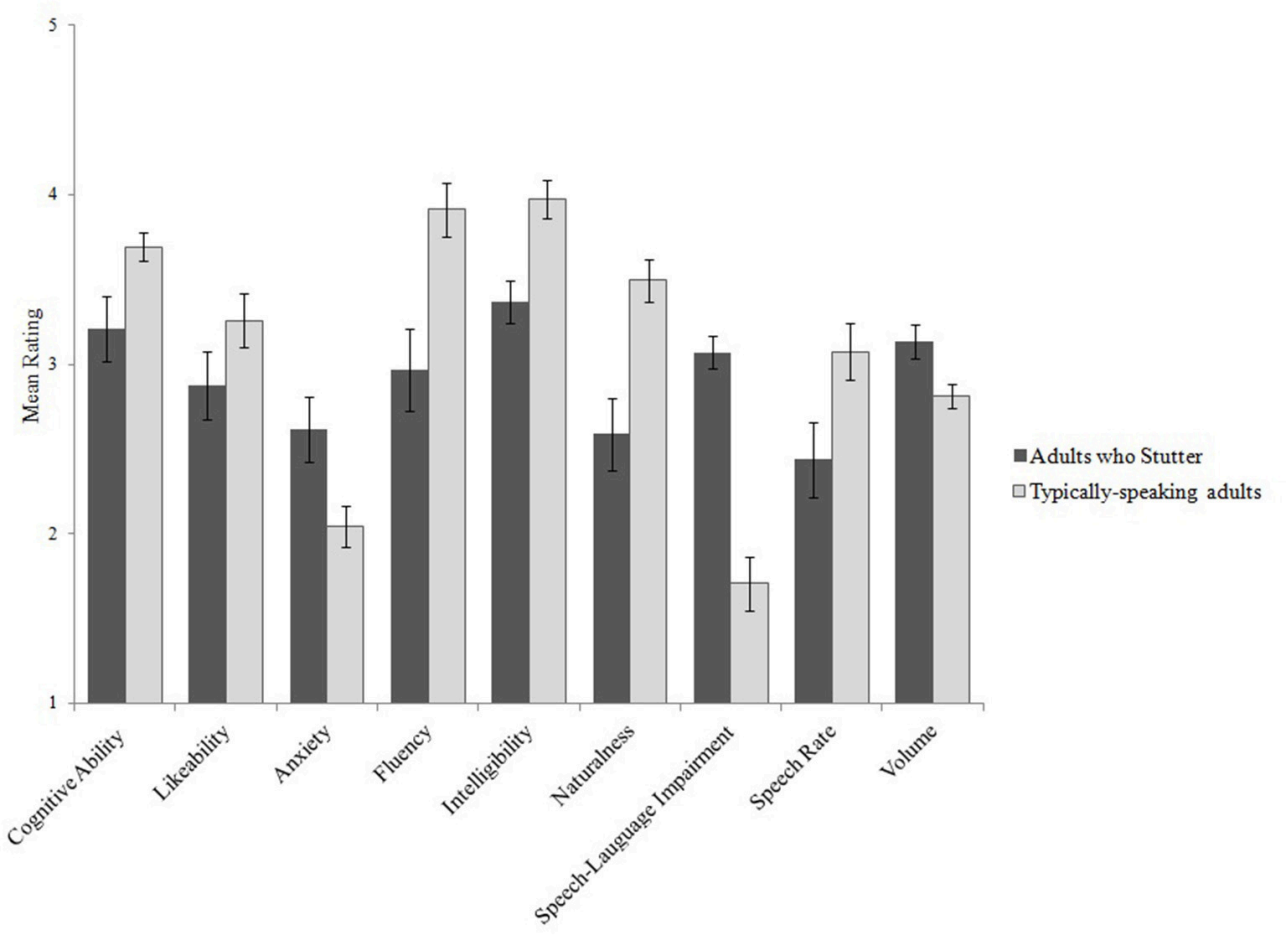
Mean ratings from Experiment 1 for each measure for adults who stutter and typically-speaking adults. Error bars indicate ±1 SEM. Higher ratings indicate greater cognitive ability, more likeable, greater anxiety, greater fluency, increased intelligibility, increased naturalness, greater likelihood of a SLI, faster speech rate, and increased volume.

Figure 1 compares mean ratings for adults who stutter and typically-speaking adult controls for each measure. Table 2 reports the ANOVA summary table with effect sizes. Overall, listeners judged the read-speech samples of adults who stutter to be less fluent, less natural, less intelligible, slower, and louder than the read-speech samples of the typically-speaking adult controls (all *p*'s < 0.05). Adults who stutter were also perceived to have lower cognitive ability, to be less likeable, and more anxious than the typically-speaking adult controls. As shown in Table 2, effect sizes (Cohen's d) for all reliable differences were large.

**Table 2:** ANOVA summary table for Experiment 1 including effect sizes.

**Adults who stutter vs. typically-speaking adults**			
**Dependent variable**	***F***_(1, 9)_	***p***	**Cohen's** ***d***
Cognitive ability	7.52^*^	0.023	0.87
Likeability	9.13^*^	0.014	0.96
Anxiety	6.68^*^	0.029	0.82
Fluency	27.45^**^	0.001	1.66
Intelligibility	17.35^*^	0.002	1.32
Naturalness	24.76^**^	0.001	1.57
Speech-language impairment	43.03^**^	<0.001	2.07
Speech Rate	25.17^**^	0.001	1.59
Volume	18.51^**^	0.002	1.36

*The table shows main effects of speaker type (adults who stutter vs. typically-speaking adults) for each of the nine measures (cognitive ability, likeability, anxiety, fluency, intelligibility, naturalness, likelihood of speech-language impairment, speech rate, and speech volume)*.

* *p < 0.05*;

** *p < 0.01*.

Next, we considered the relation between ratings of speech fluency, naturalness, intelligibility, and judged likelihood of a speech-language impairment and social or cognitive judgments about the speaker. Analyses were conducted separately for adults who stutter and the typically-speaking adult controls. Fluency, naturalness, and intelligibility were highly correlated (*r*'s > 0.9) and we created a composite *speech understanding* measure for each speaker in each group by averaging the ratings of fluency, naturalness, and intelligibility. For both adults who stutter and typically-speaking adult controls, composite speech understanding significantly predicted ratings of cognitive ability (adults who stutter: *r* = 0.91, *p* = 0.002; controls: *r* = 0.98, *p* = < 0.001), likeability (adults who stutter: *r* = 0.89, *p* = 0.003; controls: *r* = 0.95, *p* < 0.001), and anxiety (adults who stutter: *r* = −0.80, *p* = 0.02; controls: *r* = −0.73, *p* = 0.04). With respect to judged likelihood of speech-language impairment, we observed a similar, but not identical pattern. For both adults who stutter and typically-speaking adults, judged likelihood of a speech-language impairment significantly predicted cognitive ability (adults who stutter: *r* = −0.73, *p* = 0.04; controls, *r* = −0.81, *p* = 0.02) and anxiety (adults who stutter: *r* = 0.80, *p* = 0.02; control, *r* = 0.96, *p* < 0.001). Ratings of speech-language impairment were negatively correlated with likeability for both groups (adults who stutter: *r* = −0.67, *p* = 0.07; controls: *r* = −0.79, *p* = 0.02), but only reliably so for typically-speaking adult controls (*p* < 0.05).

Next we considered the relation between judged acoustic characteristics of speech rate and volume and social and cognitive impressions of the speakers in each group. For speech rate, the direction of the correlations was the same for both groups for each measure, but was not in all instances statistically reliable (α = 0.05); *p*-values are reported for all correlations. In general, individuals perceived to have faster speech were judged to have greater cognitive ability (adults who stutter: *r* = 0.68, *p* = 0.06; control: *r* = 0.90, *p* = 0.002) and to be more likeable (adults who stutter: *r* = 0.86, *p* = 0.007; controls: *r* = 0.92, *p* = 0.001). The relation between speech rate and anxiety was less clear. Individuals with faster speech tended to be judged as less anxious than individuals with slower speech, but not reliably so for either group (adults who stutter: *r* = −0.46, *p* = 0.25; controls, *r* = −0.68, *p* = 0.06). For speech volume, the only reliable relationship was between speech volume and likeability. Individuals with higher ratings of speech volume were generally judged to be more likeable (adults who stutter: *r* = 0.78, *p* = 0.022; controls: *r* = 0.74, *p* = 0.04).

Finally, we used step-wise regression to predict ratings cognitive ability, anxiety, and likeability, entering speech rate, speech volume, and %SLDs. %SLDs was entered to assess to what extent measured stuttering-like disfluencies directly predicted ratings of the social and cognitive measures. For cognitive ability, speech rate accounted for 59% of the variance in ratings with no other factors contributing additional explanatory power. For anxiety, speech volume and %SLD together accounted for 73% of the variance in ratings. For likeability, speech rate and volume together accounted for 84% of the variance in ratings.

One question that arises is whether the negative social and cognitive impressions of the read speech samples of the adults who stutter may have been primed by asking listeners questions that alerted them to the possibility that some of the speakers may have had a speech-language impairment, thereby negatively biasing their judgments. To address this possibility, a second experiment was conducted where we omitted the questions about speech fluency, naturalness, intelligibility, and whether or not the speaker had a speech-language impairment, and asked listeners only to make judgments about cognitive ability, likeability, anxiety, speech rate, and volume. All other aspects of Experiment 2 were the same as Experiment 1.

## Experiment 2

### Methods

#### Participants

A new group of ten undergraduate students (3 males, 7 females) participated in Experiment 2 in return for course credit. All participants were native English speakers who were at least 18 years of age. All participants provided written informed consent prior to participating in the experiment, in accordance with the approved procedures of the Institutional Review Board of Michigan State University. Experiment design, stimuli, and procedures were the same as Experiment 1. Experiment 2 differed from Experiment 1 only in the set of questions given to listeners.

#### Stimuli and apparatus

The same as Experiment 1.

#### Procedure

The same as Experiment 1, except for the set of questions given to participants after listening to each read-speech sample. In order to eliminate the possibility that negative social and cognitive impressions in Experiment 1 were primed by questions that led listeners to identify particular speakers as having a speech-language impairment, we omitted the questions that asked listeners to rate speech fluency, naturalness, intelligibility, and how likely they thought it was that the speaker had a speech-language impairment. All other questions were the same as Experiment 1. Listeners made ratings about speaker cognitive ability, likeability, and anxiety, as well as speech rate and volume.

#### Data analysis

The reliability of ratings across listeners for each of the rating measures were assessed through intra-class correlation coefficient (ICC). The ICC values ranged from α = 0.76 to α = 0.93. As in Experiment 1, separate within-subjects ANOVAs were conducted for each dependent measure, comparing adults who stutter to typically-speaking adults, averaging the two ratings for the two read-speech samples from each speaker.

### Results and discussion

**Figure 2:**
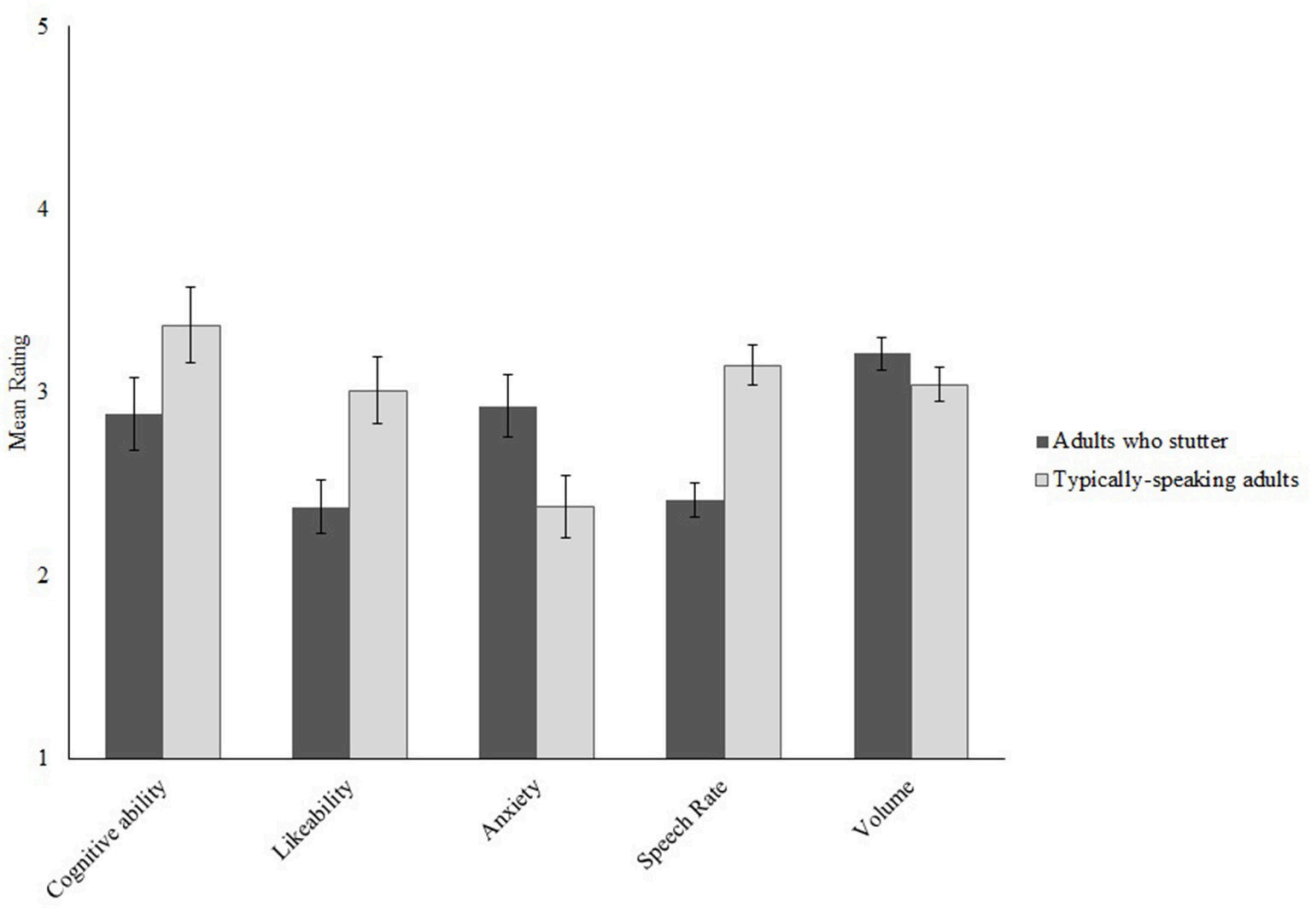
Mean ratings from Experiment 2 for each measure for adults who stutter and typically-speaking adults. Error bars indicate ±1 SEM. Higher ratings indicate greater cognitive ability, more likeable, greater anxiety, faster speech rate, and increased volume.

Figure 2 compares mean ratings for adults who stutter and typically-speaking adults for each measure. Table 3 reports the ANOVA summary table with effect sizes. Identical to Experiment 1, the adults who stutter were perceived to have lower cognitive ability, to be less likeable, and more anxious than the typically-speaking adults. The speech of adults who stutter was also judged to be slower and louder than typically-speaking adults. Overall, this pattern of results provides support that the negative social and cognitive impressions formed by participants in Experiment 1 about adults who stutter were not due to cuing participants that some of the speakers had a speech-language impairment by asking them to rate speech fluency, naturalness, intelligibility, and the likelihood that the speaker had a speech-language impairment.

**Table 3:** ANOVA summary table for Experiment 2 including effect sizes.

**Adults who stutter vs. typically-speaking adults**			
**Dependent variable**	***F***_**(1, 9)**_	***p***	**Cohen's** ***d***
Cognitive ability	6.70^*^	0.03	0.82
Likeability	10.75^**^	0.01	1.04
Anxiety	13.26^**^	0.005	1.15
Speech rate	21.70^**^	0.001	1.47
Volume	5.37^*^	0.046	0.73

*The table shows main effects of speaker type (adults who stutter vs. typically-speaking adults) for each of the five measures (cognitive abilty, likeability, anxiety, speech rate, and speech volume)*.

* *p < 0.05*;

** *p < 0.01*.

## General discussion

Previous research has shown that individuals who stutter are subject to negative perceptions unrelated to stuttering. In this study, we considered whether negative social and cognitive impressions of adults who stutter are present when naïve listeners are asked to form impressions about speakers from read-speech samples without any knowledge about the speakers. In two experiments, listeners heard samples of read speech produced by adults who stutter and typically-speaking adult controls and made judgments for each speech sample along a number of dimensions that ranged from characteristics of the speech to social and cognitive impressions of the speaker. Read-speech samples from adults who stutter were found to be less fluent, less natural, and less intelligible than the read-speech samples from the typically-speaking adults controls (Experiment 1), as well as spoken slower and louder (Experiments 1 and 2). Moreover, adults who stutter were judged to have lower cognitive ability, to be less likeable, and more anxious than the typically-speaking adult controls (Experiments 1 and 2). Critically, negative social and cognitive impressions of adults who stutter were found to hold when we eliminated questions that may have primed listeners that some of the speakers had a speech-and-language impairment (Experiment 2).

Correlation analyses considered the relation between perceptions of speech characteristics and social and cognitive impressions of the speakers, with analyses conducted separately for each speaker group in order to eliminate the possibility that reliable correlations would be driven by the overall group differences. We found that reduced speech understanding (composite measure based on ratings on fluency, naturalness, and intelligibility) and greater perceived likelihood of speech-language impairment were associated with judgments of reduced cognitive ability, reduced likeability, and greater anxiety for both speaker groups. Thus, the observed relations between speech characteristics and social and cognitive judgments were not driven by the speaker group differences and were not specific to adults who stutter, but represented a more general phenomenon that we found extended to typically-speaking adults.

At first glance, one seemingly conflicting aspect of the data is that although the between-group comparisons in loudness and likeability reveal that adults who stutter generally talk more loudly than the typically-speaking adult controls and are perceived to be less likeable, the correlations within each group are in the opposite direction; individuals who speak more loudly are perceived to be more likeable. What we believe this suggests is that the overall lower likeability ratings for adults who stutter is being mediated by perceived loudness. That is, the adults who stutter are perceived to be less likeable than the typically-speaking adults, but speaking more loudly reduces this group difference; within the stuttering group, the adults who stutter who also speak the loudest tend to be adults who stutter who are the most likeable.

One question that emerges from this work is whether the individual differences in speech characteristics reflected actual differences in cognitive ability. To assess this possibility, we considered the relation between speech characteristics and an operation span measure of working memory, which we used as a proxy for cognitive ability. Past research has shown that the operation span task is a strong predictor of fluid reasoning abilities (Unsworth and Engle, 2005), and is correlated with other working memory measures (Unsworth et al., 2005). None of the speech characteristic measures were found to be related to the operation span measure (all *p*'s > 0.35), supporting the conclusion that the observed relationships between speech characteristics and judged cognitive ability were not being driven by differences in actual cognitive ability.

Overall, the results of the present study add to the body of work showing individuals who stutter are subject to broad negative social and cognitive impressions (e.g., Woods and Williams, 1976; Woods, 1978; St. Louis and Lass, 1981; Doody et al., 1993; Gabel, 2006). The present study is the first to show that negative social and cognitive impressions are formed about adults who stutter by lay listeners from samples of naturally-read speech without any knowledge about the speakers producing the samples. In both experiments, adults who stutter were judged to have lower cognitive ability, to be less likeable, and to be more anxious than typically speaking adults.

One question that arises is whether differences between groups could have emerged because the differences in the speaker groups may have been obvious in the read speech samples. In our view, this seems unlikely for several reasons. First, the percentage of speech disfluencies for the read-speech samples from the adults who stuttered was only 2.4%, with the usual cut off to be categorized as a stuttering person is 3% stuttering-like disfluencies per 100 syllables. Thus, in the present study, the listeners who made judgments about the read-speech samples were both not provided any information about the individuals producing the samples and difference between the two groups of speakers were more subtle than they would likely be in conversational speech. Second, the same general group differences were observed in Experiment 2 when we did not ask listeners to judge aspects of the speech that may have primed listeners that some of the speakers had a speech-and-language impairment. Third, the present study shows that relations between speech characteristics and social and cognitive impressions of the speakers tend to be found for *both* groups of speakers when either the speech samples of each group are considered separately or combined. Finally, one of the most predictive cues identified in the present study was speech rate. Faster speech is associated with greater cognitive ability and increased likeability, and to a lesser degree with decreased anxiety. Correlations between speech rate and anxiety are the weakest and not reliable for either group. Speech volume accounts for unique variance in both likeability ratings and anxiety ratings. Notably, perceived anxiety was the only speaker characteristic of the three measured where percentage of speech-language disfluencies contributed significantly to ratings above and beyond speech rate and speech volume.

Overall, these findings highlight that the acoustic characteristics of a person's speech can significantly influence social and cognitive impressions of that person, separate from the impressions that we form based on other cues. Moreover, potentially subtle acoustic differences in produced speech can lead to negative social and cognitive perceptions for both individuals with speech-language impairments and more fluent-speaking adults. These findings are more broadly consistent with work that has shown that negative perceptions of individuals can arise when listeners are asked to form social impressions based on the speech of individuals that differs from their own speech, such as with foreign-accented speech or speech from a different dialect (Markel et al., 1967; Gallois and Callan, 1981; Lev-Ari and Keysar, 2010). One factor that we did not consider in the present study is the potential for speaker gender effects or interactions between speaker gender and the various measures considered. Most chronic adult stutterers are male with our sample of speakers reflecting this gender difference. Further work is needed to assess the potential for gender effects. More broadly, additional studies are needed to identify the contribution of a broader range of acoustic characteristics of speech in the formation of social and cognitive impressions for individuals with and without disordered speech.

## Author contributions

LA, JDM, SC, and JW were involved in the concept and design of the experiments. LA was involved with data collection. LA, JDM, SC, and JW were involved with data interpretation. LA and JDM were involved with data analysis and drafting. LA, JDM, SC, and JW were involved with revisions to the manuscript and approved the final version.

### Conflict of interest statement

The authors declare that the research was conducted in the absence of any commercial or financial relationships that could be construed as a potential conflict of interest.
