# Supplementary material for: Social and Cognitive Impressions of Adults Who Do and Do Not Stutter Based on Listeners' Perceptions of Read-Speech Samples
Source: Front Psychol. 2017 Jul 11;8:1148. doi: 10.3389/fpsyg.2017.01148 (PMC5504238; doi:10.3389/fpsyg.2017.01148)
Supplement: Supplementary file 1 [file DataSheet1.docx]

**Appendix**

Passages for read-speech samples

The Age of Exploration lasted from the early fifteenth century until the middle of the seventeenth century. During this time period, European nations explored the world by sea. They wanted to trade goods with other people to make a profit. They also wanted to find shorter routes to their existing trade partners. Some adventurers wanted to find out information about the world. Cartographers were constantly revising maps based on the latest travels. Through the maps and journals of explorers, people of the known world improved their knowledge of geography. Spain was the first European nation to claim land in the Americas.

*Syllables: 166*

You might think that all living things are classified as either plants or animals, but there are some mysterious little organisms which are neither, yet are still alive. Many are invisible, hiding deep in the ground or floating silently on the air. Unlike plants, they do not rely on the heat or light of the sun for survival. They must find a source of nutrients outside themselves. They are very adaptable to any weather conditions. If temperatures fall too low to support life, they go into a deep sleep. This sleep is like the hibernation state that some animals use during the coldest part of the winter.

*Syllables: 159*

Your body is approximately sixty percent water. Water is part of all the cells in your body and the plasma in your blood. Water helps your cells receive the nutrients they need, and it helps take away the wastes. All living things produce wastes. It is the job of the excretory system to regulate the amount of water that you have in your body and to help remove wastes from your system. If wastes build up in your blood and in your cells, your body becomes toxic, which can be deadly. Several different organs are involved in your excretory system. Even your skin participates in the process!

*Syllables: 153*

Native American Indian groups in North America lived in different cultural regions, each of which developed its own customs and traditions. A custom is the specific way in which a group of people does something. This can include how foods are prepared, what clothing is worn, the kinds of celebrations and much more. The set of customs developed and shared by a culture over time is a tradition. A culture’s customs are often determined by the natural resources found in their environment. In the Desert Southwest region, cloth weaving developed as a custom. The area has fewer large animals whose skins can be used for making clothing or blankets.

*Syllables: 168*

People are consumers. We have to spend large parts of our days finding, buying, cooking and eating our food. Did you ever think it might be nice to be able to make your own food like plants do? Plants are producers and perform a process called photosynthesis using light from the sun, water and carbon dioxide. Carbon dioxide is the gas we exhale when we breathe. The end result of this chemical reaction is sugar for the plant to “eat.” The plant releases water and oxygen, a gas all animals need to breathe, into the air. Our quality of life and the very quality of the air we breathe depends upon our green plant partners.

*Syllables: 163*

Many historians and scientists believe that the earliest people in North America may have traveled here from the continent of Asia many thousands of years ago. At that time, Earth was experiencing an Ice Age. Much of the water that separates the northern parts of Asia and North America would have been frozen at the time. It may have formed an ice bridge that people were able to walk across. Perhaps they were hunters following their food. Perhaps they were adventurous and wanted to explore. We do not know for sure. Their migration to North America, however, may make them the ancestors of the people we call Native Americans.

*Syllables: 169*

Any fraction that has the same number as its numerator and denominator is equal to one. The denominator tells how many equal parts the whole has been divided into, and the numerator tells how many of those parts you have. You could cut something into a million parts. As long as you keep all of those parts, you still have a whole. Because we can have fractions that are equal to one, we can use them to help create equivalent fractions. We find common denominators when we add or subtract fractions. We then simplify fractions when we write our final answer. When you multiply or divide by a fraction that is equal to one, the value does not change.

*Syllables: 154*

The circulatory system is the transport system of the human body. Your body is like a map filled with passageways of different sizes that are filled with blood. At the very center of the circulatory system is the heart. Your heart is about the same size as your fist, but it is made of muscle. Its job is to pump your blood through all those blood vessels. It never stops working, even when you are sleeping. It is the strongest muscle in your body. Your heart has four chambers, or spaces, inside it. Each chamber is separated by a valve that allows blood flow in only one direction.

*Syllables: 150*
